# Functionally Diverse NK-Like T Cells Are Effectors and Predictors of Successful Aging

**DOI:** 10.3389/fimmu.2016.00530

**Published:** 2016-11-24

**Authors:** Joshua J. Michel, Patricia Griffin, Abbe N. Vallejo

**Affiliations:** ^1^Department of Pediatrics, University of Pittsburgh School of Medicine, Pittsburgh, PA, USA; ^2^Children’s Hospital of Pittsburgh, University of Pittsburgh School of Medicine, Pittsburgh, PA, USA; ^3^Department of Immunology, University of Pittsburgh School of Medicine, Pittsburgh, PA, USA; ^4^Pittsburgh Claude Pepper Older Americans Independence Center, University of Pittsburgh School of Medicine, Pittsburgh, PA, USA

**Keywords:** CD16, CD56, cell senescence, functional performance, immune remodeling, NKG2D, plasticity, TCR-independent

## Abstract

The fundamental challenge of aging and long-term survivorship is maintenance of functional independence and compression of morbidity despite a life history of disease. Inasmuch as immunity is a determinant of individual health and fitness, unraveling novel mechanisms of immune homeostasis in late life is of paramount interest. Comparative studies of young and old persons have documented age-related atrophy of the thymus, the contraction of diversity of the T cell receptor (TCR) repertoire, and the intrinsic inefficiency of classical TCR signaling in aged T cells. However, the elderly have highly heterogeneous health phenotypes. Studies of defined populations of persons aged 75 and older have led to the recognition of successful aging, a distinct physiologic construct characterized by high physical and cognitive functioning without measurable disability. Significantly, successful agers have a unique T cell repertoire; namely, the dominance of highly oligoclonal αβT cells expressing a diverse array of receptors normally expressed by NK cells. Despite their properties of cell senescence, these unusual NK-like T cells are functionally active effectors that do not require engagement of their clonotypic TCR. Thus, NK-like T cells represent a beneficial remodeling of the immune repertoire with advancing age, consistent with the concept of immune plasticity. Significantly, certain subsets are predictors of physical/cognitive performance among older adults. Further understanding of the roles of these NK-like T cells to host defense, and how they integrate with other physiologic domains of function are new frontiers for investigation in Aging Biology. Such pursuits will require a research paradigm shift from the usual young-versus-old comparison to the analysis of defined elderly populations. These endeavors may also pave way to age-appropriate, group-targeted immune interventions for the growing elderly population.

## Introduction: Alterations in Classical T Cell-Mediated Immunity During Aging

Studies comparing young and old humans and mice have led to a voluminous body of literature showing a general age-related decline in various physiologic functions. In the immune system, among the most notable age-dependent physiologic retrogressions in the T cell compartment are inefficiencies in classical T cell receptor (TCR) signaling, thymic involution, contraction of the naïve T compartment, expansion of the memory T cell compartment, and overall shortening of telomeres (1–8). At the cellular level, aged CD4^+^ and CD8^+^ T cells have a deficiency in the expression of CD28 that coincides with highly shortened telomeres, high levels of expression of mitotic inhibitors, such as p16 and p53, and a severe limitation or complete lack of mitotic activity (9–13). All of these alterations have been argued to underlie the relative poorer antigen-specific T cell-dependent immunity among older adults compared to younger persons.

## Heterogeneity of Phenotypes of Older Adults

Older adults (generally defined as those aged ≥65 years), however, have highly heterogeneous health and immune phenotypes. They range from the frail and chronically ill residents of long-term care facilities to the community dwellers that are living independently (14–17). Many of them retain their ability to mount vaccine responses, including to the pandemic and seasonal influenza vaccines, and to the zoster vaccine (18–22). There are evidences of functionally active virus-specific T cells during new and reactivated latent infections (23–25). Old age has also become less of hurdle in the setting of organ transplantation for either organ donors or recipients (26–29). Thus, aging is not synonymous with poor health, or that the elderly are not mere defective versions of the young.

Heterogeneity of older adults provides a compelling rationale for a re-appraisal of “immunosenescence.” In its current usage, the term refers to the poorer degree of immune responsiveness of older adults relative to that seen in the young, a generalized and vague definition that has not substantially differed from the original concept proposed by Walford in the 1950s (30). Learning from epidemiological and geriatric studies (14–17, 31), we have articulated the paramount importance for the analysis of defined populations of the elderly, instead of continuing with the usual young-versus-old comparative approach. Such research paradigm shift is a key toward unraveling immunopathways that underlie discrete physiologic constructs of aging, such as frailty and successful aging (32, 33).

## Irreversible Loss of CD28: A Signature of Aging in Human T Cells

CD28 is the major co-stimulatory molecule that is required to sustain normal T cell activation (34) and for the elaboration of antigen-specific effector function in both naïve and memory compartments (35–37). In cohort studies, we provided the definitive proof for progressive loss of CD28 with chronologic aging (12). Such loss or absence of CD28 has long been thought to lead to deficiency or inefficiency of TCR signaling in aged T cells (10, 38). Indeed, mice with homozygous deletion of *CD28* results in an immunosuppressed phenotype, since mouse *CD28*^−/−^ T cells are anergic and prone to activation-induced cell death (35, 39).

The loss of CD28 on human T cells with aging (10, 12, 40) may not be surprising since CD28 expression is subject to transient downregulation during a normal immune response (41). In fact, deficiency of its expression is characteristic of continuous passages of T cell cultures (40, 42). These unusual CD28^null^ CD8^+^ T cells have shortened telomeres (13), consistent with telomere-dependent senescence (sometimes referred to as “replicative senescence”) akin to those reported for other human somatic cells (43–47).

Due to more rapid turnover, CD8^+^ T cells have higher rate of CD28 loss than CD4^+^ T cells (48, 49). CD28^null^ CD4^+^ and CD8^+^ T cells are highly oligoclonal and have highly shortened telomeres, indicating their long replicative history (12, 13). They also have high expression levels of p16 and 53, and they have limited, if not complete lack of, proliferative capacity even under conditions of optimal stimulation *via* TCR/CD3 in the presence of interleukin (IL)-2 *in vitro* (11, 12, 50, 51). All these properties are consistent with replicative senescence.

CD28 loss and telomere shortening are properties of primates, being typical of elderly humans as described above, as well as for older macaques and other anthropoids (52–55). In contrast, mouse T cells maintain long telomeres, and neither CD4^+^ nor CD8^+^ T cells show perceptible telomere shortening with multiple cell divisions *in vitro* (56). Indeed, it takes at least four generations for the telomerase-deficient mouse to show quantitative shortening of telomeres (57), indicating mice clearly do not undergo telomere-dependent replicative senescence.

Clonal expansions of T cells are characteristic of old mice similar to old humans (58). However, mouse T cells do not lose CD28 expression with chronologic aging. In fact, CD28 expression level may actually increase with age (59). Such species-specific difference in CD28 expression pattern between humans and mice is attributable to entirely non-homologous DNA sequences in the promoter regions of the *CD28* gene (60) (*Homo sapiens* CD28, NCBI Gene 940, HGNC 1653; *Mus musculus* CD28, MGI 88327, NCBI Gene 12487). These age-related loss/maintenance of telomeres and loss of CD28 underscore that transposition of data obtained from mouse studies to human biology is unsound. We have articulated that while aging mouse models are instructive about the general biology of aging, they do not substitute for analytical studies of human elderly subjects (61).

The loss of CD28 is generally irreversible, due to the direct inactivation of the gene promoter (42, 62). The transcriptional initiator, a DNA sequence module in the 5′ *cis*-acting *CD28* regulatory region where the activator complex, including nucleolin and heterogeneous ribonucleoprotein-DOA, is unoccupied in senescent CD28^null^ T cells (63). Nucleolin and heterogeneous ribonucleoprotein-DOA are found in senescent T cells, but they do not form a functional initiator complex. While mechanism(s) underlying the failure of the assembly of this transcriptional complex remains to be investigated, it is clear that non-occupancy of the *CD28* initiator results in a transcriptional block, leading to the absence of all splice forms of *CD28* mRNA and the lack of expression of CD28 on the T cell surface (42, 64, 65).

CD28^null^ T cells are resistant to apoptosis (66), which explains their persistence in circulation for years and their pervasive accumulation *in vivo* with advancing age. This is attributed to constitutively high levels of expression of Bcl2 and Bcl-xL, with corresponding downregulation of Bax (12, 67). Bcl-independent pathways for the lifelong persistence of these cells have also been reported (68).

## *De Novo* Expression of NK-Related Receptors on CD28^null^ T Cells: Functional Diversity and Versatility of Aged T Cells

Whether they are naturally derived *in vivo* during aging, or in an *in vitro* senescence system, oligoclonal senescent CD28^null^ T cells have a unique phenotype for their *de novo* acquisition of a diverse array of receptors normally expressed on NK cells (12, 50, 69, 70). The repertoire of NK-related receptors they express does not reflect the full complement of the many *NK receptor* genes normally expressed on NK cells (50). However, the NK-related receptors on aged CD28^null^ T cells are expressed co-dominantly in varying combinations along clonal lineages. CD28^null^ T cells with identical *TCR CDR3* sequences, indicating their common origin from a single mother CD28^+^ T cell, may express different types of NK-related receptors (71, 72).

Whether the loss of CD28 is required for, or is an event independent from, the expression of NK-related receptors remains to be examined. However, it is clear that differences in the patterns of expression of these receptors between NK cells and CD28^null^ T cells are related to cell-specific differences in the regulatory modules of each NK-related receptor. For example, we have shown that differential expression of CD158b1 (KIR 2DL2) between T and NK cells are controlled by two distinct transcriptional regulatory motifs on the upstream *cis*-acting promoter region of the gene; namely, a proximal element at −51 and an AML site at position −98 for T and NK cells, respectively (73). Other investigators have reported the role of age-related epigenetic alterations. Differential induction of CD158d (KIR 2DL4) and CD158b2 (KIR 2DL3) on T cells is related to methylation/demethylation on promoter regions of these two genes, in contrast to their classical promoter-driven expression as seen in NK cells (74–76). These studies suggest that there may be diverse regulatory machineries involved in the induction of NK-related receptors on T cells with aging. Given the diversity of these receptors and their apparent co-dominant expression, it will be of interest to examine whether and how expression of one NK-receptor affects the expression of another NK-receptor during the aging process. A particular interest is the regulation of expression of the prototypic receptors CD56, CD16, and NKG2D on aged T cells. But regardless of whether such regulation occurs at the level of unique promoter motifs, or through structural alterations of chromatin that favor accessibility of the particular *NK-receptor* gene, or perhaps through posttranscription controls, it is clear that the acquisition of NK receptors by T cells corresponds with the elaboration of new effector function (77).

The phenomenal age-related expression of NK-related receptors on T cells has been associated with seropositivity to cytomegalovirus (CMV) (78). This is in line with reports about similar association of CMV serology with frequency of CD28^null^ T cells, and such serological-cellular association has been argued to be a predictor of poor health outcomes of aging (79, 80). It has also been suggested that CMV infection may lead to the emergence of these senescent T cells that are considered dysfunctional or non-functional (81–83). However, such studies are purely associational rather than causal. Further, the association is not universal. The cited studies are mostly from those on elderly populations in Northern Europe where CMV exposure appears to occur gradually over the life span, which might explain the high CMV seropositivity in old age (80). In the United States, CMV exposure is already widespread at early adolescence (84). Yet, we have shown that senescent NK-like T cells are rarely found among young Americans (12, 32). Importantly, we found very high titers of anti-CMV antibody among older adults and found no clinical evidence of CMV disease. Indeed, another cohort study showed CMV seropositivity alone is an insufficient measure of health risk among older Americans (85). In addition, populations of CMV-specific T cells have been found to be functional with clear beneficial antiviral effects (68, 86, 87). A recent experimental study has shown further that CMV by itself does not induce replicative senescence for T cells (25). CMV disease is undoubtedly serious whether it happens at an early or old age. However, the causative role of CMV in human T cell senescence is yet to be proven. Broader experimental studies are needed to determine what particular environmental and/or endogenous factors trigger, drive, and maintain populations of senescent NK-like T cells *in vivo* during the aging process.

**Figure 1:**
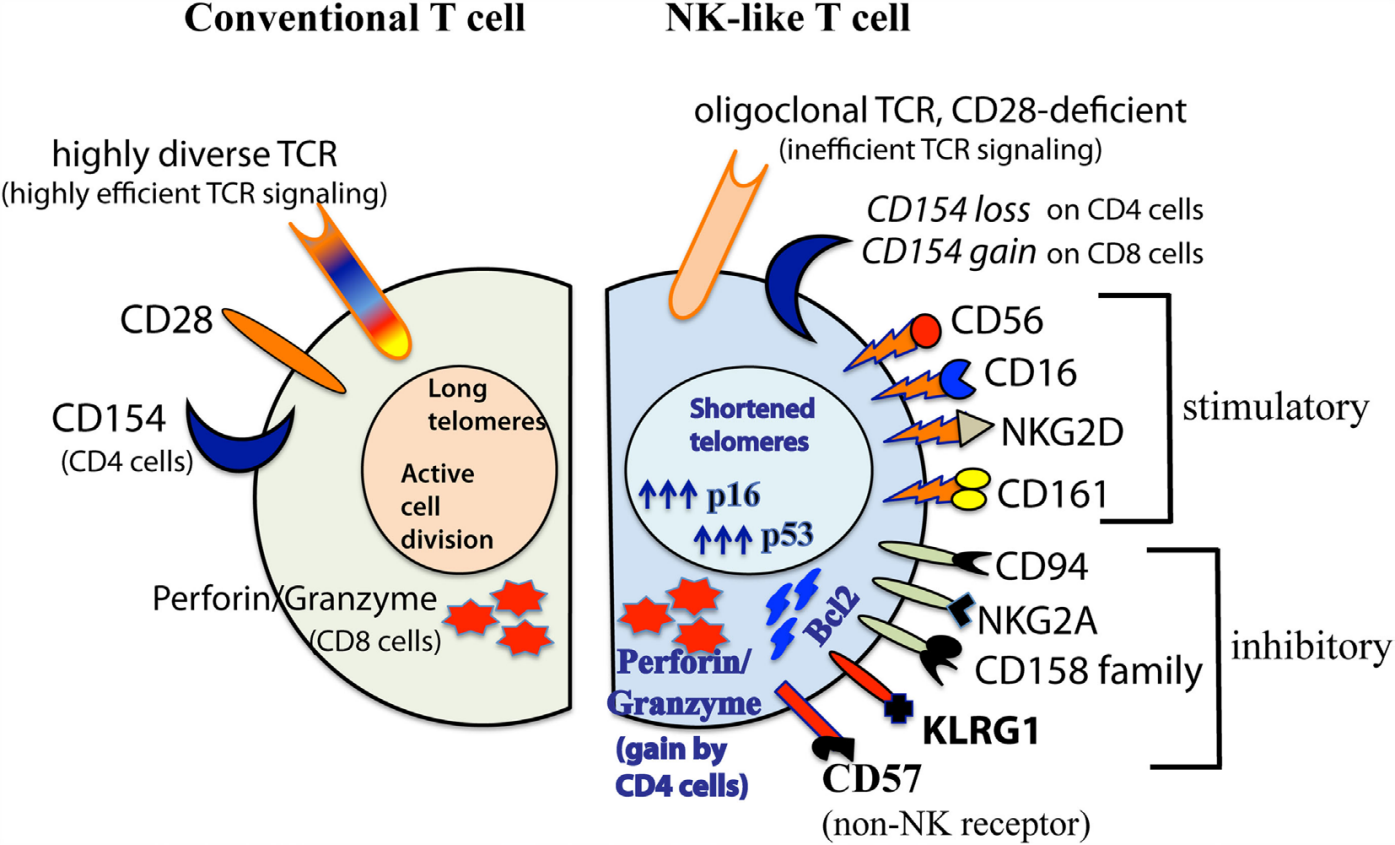
**Diagrammatic comparison between conventional CD28^+^ and senescent CD28^null^ NK-like T cells**. This illustration summarizes findings from various investigators as described in the text. The diagram shown was modified from (32) with permission from *Aging and Disease* journal under the terms of Creative Commons Attribution License (CC BY). This license allows the unrestricted use, distribution, modification, and reproduction in any medium by the author with credited citation of the original publication.

The array of NK-related receptors expressed on aged CD28^null^ T cells is summarized in Figure 1. They include the prototypic stimulatory NK receptors, CD16, CD56, and NKG2D. They may also express CD161, and various inhibitory NK receptors such as CD94 and NKG2A, and members of the CD158 killer cell immunoglobulin-like receptor family (12, 50, 69, 72, 77, 88–90). Unlike the selective single allelic expression for TCR, NK-related receptors are expressed co-dominantly on aged T cells.

In addition to shortened telomeres, high p16/53 expression levels, and irreversible loss of CD28, aged NK-like T cells express two other markers of senescence, namely, KLRG1 and CD57 (81, 91, 92). KLRG1 is an inhibitory NK-related receptor that has been shown to actively suppress classical TCR signaling (93). CD57 is an adhesion molecule that is typically expressed on terminally differentiated T cells. Although it is still unclear if CD57 itself is a signaling receptor that dictates or alters T cell effector function, its expression on T cells is biomarker for cell cycle arrest in aged T cells (91, 94). It might be noted that CD57 is also expressed on highly differentiated NK cells (95, 96). However, whether such CD57^+^ NK cells are senescent, and that CD57 directly controls NK cell function are also not yet known.

Despite their senescent properties, CD28^null^ NK-like T cells are highly functional and versatile. While there is general trend for the varying inefficiencies of classical TCR signaling during aging (1, 2, 97–99), there could still be residual TCR signaling as exemplified by long-lived memory T cells in the context small pox and polio vaccination (100, 101). Indeed, experimental studies showing unusually high constitutive level of expression of interferon (IFN) γ in CD28^null^ T cells can further increase following ligation of TCR/CD3 (38, 94, 102). Such residual TCR-driven response may be attributed to other co-stimulatory molecules, such as 41BB ligand, OX40, CD70, and CD58, which substitutes for the defunct CD28 (103–107).

More significantly, we have reported that effector activities of CD28^null^ NK-like CD4^+^ and CD8^+^ T cells are directly attributable to signaling of the NK-related receptors they express in a totally TCR-independent manner (12, 70). We have shown that CD56-driven and NKG2D-driven expression of the early activation cell surface antigen CD69; the intracellular expression of IL-4, IFNγ, CD107b/LAMP2, perforin, and granzyme; and the late cell surface expression of exocytosis protein CD107a are to be as effective as, if not better than, classical TCR stimulation. In fact, the CD56-/NKG2D-driven TCR-independent expression of perforin, granzyme, and CD107a occur in both CD4^+^ and CD8^+^ NK-like T cells. This indicates that the conventional “helper” and “cytotoxic” designations for CD4^+^ and CD8^+^ T cells, respectively, are not instructive about of the biology of T cells in old age. Similarly, the expression of CD154 (CD40 ligand) on aged T cells does not follow the usual CD4 helper paradigm. CD154 is lost on senescent CD28^null^ CD4^+^ NK-like T cells but is gained by senescent CD28^null^ CD8^+^ NK-like T cells (38, 108, 109). This suggests that the latter cell subset is a potential target to boost humoral immunity in the elderly.

## Age-Dependent Accumulation of CD28^null^ NK-Like T Cells with Oligoclonal TCRs: Immune Repertoire Remodeling Consistent with Physiologic Plasticity in Old Age

Physiologic systems are optimized toward reproduction, after which the goal is individual survival (110). There is evolutionary conservation of biological pathways that ensure individual survival beyond reproductive maturity (111, 112), including a variety of genes referred to as “longevity assurance” genes that promote long-term survival (113–116). Older organisms are essential in maintaining population structures particularly among social animals and are therefore involved ultimately in the perpetuation of the species (112, 117–119).

Immunity is an evolutionary determinant of individual fitness and survival (120–122). The accumulation of NK-like CD28^null^ T cells with advancing age represents a remodeling of the immune repertoire as a compensatory mechanism for the general age-related losses in conventional T cell-dependent immunity (123). As described previously, there is thymic atrophy with age leading to impaired production of new naïve T cells, making older adults unable to respond to new and emerging pathogens in an antigen-specific manner (3, 124). With antigenic exposure through life, there is progressive contraction of the naïve T cell compartment, with corresponding expansion of memory and senescent T cell compartment. These events over the lifespan result in the contraction of diversity of the clonotypic TCR repertoire (5, 49). With cycles of expansion and death of T cells during antigenic challenges, the phenomenal accumulation of apoptosis-resistant CD28^null^ NK-like T cells is likely a protection against clinical lymphopenia, which is very rare among older adults (125, 126).

The acquisition of a diverse array of NK-related receptors on CD28^null^ T cells maintains immunologic diversity in old age. As discussed previously, there is co-dominant expression of diverse NK-related receptors along clonal lineages of CD28^null^ T cells in late life. This is in stark contrast to the conventional clonotypic TCR diversity that is characteristic of the young. Signaling of these NK-related receptors effectively imparts an innate function to aged T cells (12, 70); hence, we had originally introduced the term “NK-like T cells” to emphasize their NK-related receptor-driven, TCR-independent effector function (50). The term underlines the diverse array of NK-related receptors expressed along oligoclonal TCRαβ lineages, in contrast to convention αβTCR repertoire diversity in the young (12). NK-like T cells are distinct from conventional NKT cells (or invariant iNKT cells), which are identified a single invariant TCR AV24BV11 that recognizes glycolipid antigens presented in the context of CD1d instead of conventional HLA antigen-presenting molecules (127, 128).

NK-like T cells compensate for the corresponding age-related functional loses in the NK cell compartment (32). NK cell numbers are largely maintained through life, but skewing of certain NK cell subsets with aging have been reported (129). We have shown that octo-/nona-genarians have contracted pools of CD56^+^ and CD16^+^ NK cells, which are accompanied by corresponding age-dependent gains of CD56 and CD16 expression on both CD4^+^ and CD8^+^ T cells (32, 70). As already described previously, CD56 ligation alone can drive T cell effector activities. The function of CD16 on NK-like T cells remains to be examined.

Induction of NK-related receptors on T cells may not be surprising since T cells and NK cells originate from a common lymphoid progenitor. We have shown that NK cells have an abundance of untranslated, but re-arranged, *TCRαβ* mRNA with sequences identical to those seen in T cells (130). Thus, inducibility of NK-related receptors in senescent CD28^null^ NK-like T cells is consistent with functional plasticity of T cells (131–133). Although the intricacies of T cell plasticity are still being investigated, such plasticity re-directs the elaboration of effector activities to ensure a vigorous immunity. In old age, signaling of effector activities of NK-like T cells through NK-related receptors is an adaptation of the aging immune system. Such adaptation is a way to maintain immune homeostasis despite the inefficiency of classical TCR signaling and the contraction of diversity of the repertoire of clonotypic TCRs. NK-like T cells are highly resistant to cell death (12) and may represent Darwin’s “fittest” lymphocytes that contribute to immune function into old age.

Cell senescence is undoubtedly a characteristic of old organisms, and it contributes to age-related malfunction in various tissues/organs (44, 134, 135). However, cell senescence also has physiologic benefits. Among these is its role in tumor suppression (134, 136, 137). Cell senescence also plays a role in tissue repair (138), such as in the prevention of fibrosis in liver, skin, kidney, and heart, and in the prevention of atherosclerosis and pulmonary hypertension (139). In addition, there is also programed cell senescence, which is an essential component of embryogenesis (140–142). Along these lines, the age-dependent emergence of functionally competent senescent NK-like CD28^null^ T cells represents a significant and beneficial remodeling of the immune repertoire (123).

T cell repertoire remodeling through the *de novo* expression of NK-related receptors along clonal lineages of senescent CD28^null^ T cells is also consistent with age-related functional plasticity in certain organ systems. For example, there is age-related structural and functional decline in the central nervous system that leads to varying degrees of cognitive impairment, such as dementia and Alzheimer’s disease. There is heritability of high cognitive function into old age (143, 144). The roles of specific genes or gene polymorphisms, and epigenetic programs have been reported (114, 115, 119, 145–150). But the apparent “default” trajectory of age-related cognitive decline may be altered by physical activity, inclusive of regimented exercise, strength training, or usual activity such as walking. This has been best illustrated by improvement of various aspects of cognitive function, including memory and learning, among older adults engaged in regular physical activity (151–158). Functional brain imaging show extraordinary brain networks of neurocognitive performance following physical activity (159–161). In experimental animals, physical activity elicits an array of genes, along with epigenetic changes, associated with improvement in neurobehavioral performance (162–165). While the mechanisms underlying the improvement of brain/cognitive function with physical activity need to be examined further, aging of the brain is undoubtedly amenable to modulation.

Similarly, aging leads to a decline skeletal muscle function, including an age-related inefficiency of muscle mitochondria. Yet, the aging skeletal muscle is functionally plastic. Whereas certain gene polymorphisms have been implicated to maintain muscle function with age (166), physical activity has been shown to improve muscle and mitochondrial function among older adults (167–170). An important component of physical activity-induced improvement of function of the aging muscle is the equally plastic satellite cells that maintain muscle organization (171, 172). Clearly, certain physiologic systems including immune cells are functionally plastic, a property that may be exploited to maintain, if not improve, functional performance in old age.

## NK-Like T Cell Subsets are Bioindicators of Successful Aging and Longevity

As described previously, older adults are highly heterogeneous, with varying health phenotypes and life expectancy. An improved understanding of this heterogeneity has been facilitated by objective measurements of physical and cognitive function. Such measurements have led to better stratification of elders; from frail residents of long-term care facilities, to successfully aging community dwellers (16, 31, 153, 173–177). Thus, we have been proponents for the integration of immunity with other domains of function (32).

Integration of immunity to other physiologic systems may be best illustrated by our cross sectional study of the All Stars cohort (70) of the survivors from the Cardiovascular Health Study, a multicenter long-term study of aging (16, 178, 179). Categorization of elders was based on cognition scores (3MS), measured by the average of three tests using the modified minimental examination (180), and self report of difficulty in performing activities of daily living (ADL), namely, dressing, toileting, transferring, eating, and bathing (181). High functioning (or “unimpaired”) was defined as 3MS score >80 and ADL = 0. The data showed that the stimulatory NK-related receptors CD16, CD56, and NKG2D in all T cell subsets were the most prominent cellular components of the immune signature of the high functioning group as determined by factor analysis. In contrast, the inhibitory NK-related receptors NKG2A, CD158a, and CD158e comprised the cell signature of the functionally impaired. In line with these fingerprints, logistic regression analysis of the same dataset showed CD56 and CD16 expression was significant predictors of high functional performance. In contrast, NKG2A and CD158a were negative predictors. More importantly, CD28^null^ T cells in the CD4 but not in the CD8, compartment expressing these four NK-related receptors were the cell subset predictor of high cognitive/physical functioning.

**Figure 2:**
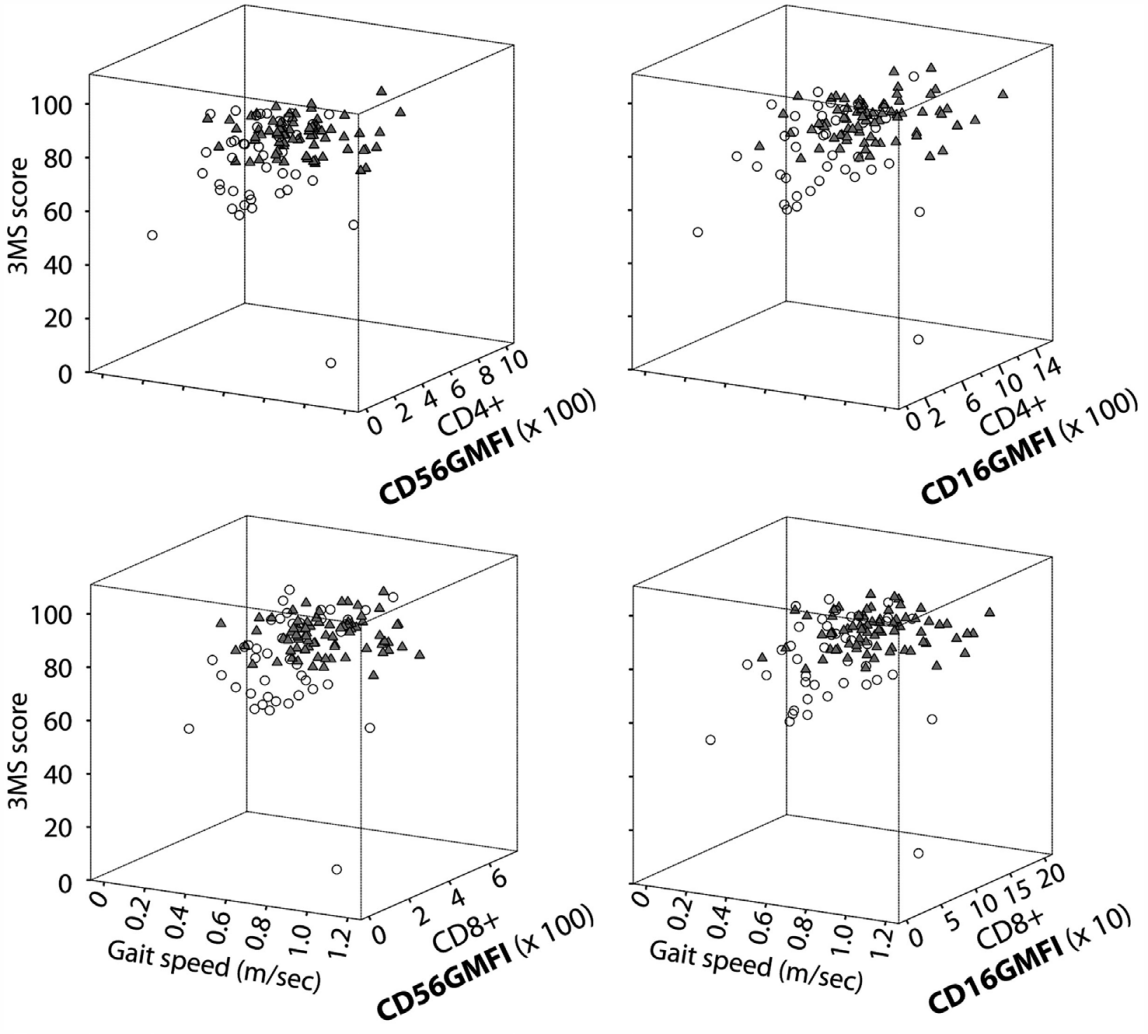
**NK-like T cells are linked to high cognitive and physical function**. Data shown are 3D scatter plot summaries from the re-analyses of our data from the All Stars cohort of the Cardiovascular Health Study (70). CD16 and CD56 expression on CD4^+^ CD28^null^ and CD8^+^ CD28^null^ T cells are expressed as GMFI, which was determined by multicolor flow cytometry. Older adults were grouped as unimpaired (*solid triangles*) or impaired (*open circles*) based on a simple criterion of ADL = 0 and ADL > 1, respectively. Measurements of 3MS cognition score and gait speed and ADL scoring are as described in the text.

Another way to illustrate the relationship between NK-like CD28^null^ T cells and physical/cognitive functioning is shown in Figure 2 with a three dimensional plot for CD16 or CD56 expression levels (measured as GMFI, geometric mean fluorescence intensity), 3MS cognition score, and gait speed. The latter measure of physical function was determined by a 4-m walk test that has been standardized/validated from various cohort studies (16, 176). The data show a clear segregation between the high functioning and functionally impaired elders. This is surprising given that “impaired” and “unimpaired” categories in this graphical illustration are very loosely defined by ADL ≥ 1 and ADL = 0, respectively. Therefore, it will be of significant interest to determine if this three-way relationship between subsets of NK-like T cells, physical function, and cognitive ability translates into vigorous immune defense. In addition, the underlying mechanistic link(s) between these three physiologic systems will be instructive about integrative physiology of successful aging.

## NK-Like T Cells in Young Persons with Chronic Diseases: A Case for Antagonistic Pleiotropy

NK-like CD28^null^ T cells represent a beneficial remodeling of the T cell repertoire with aging. Paradoxically, similar cells have also been found among young patients with chronic immune-mediated diseases in an age-disproportionate manner. We have shown the infiltration of CD56^+^ CD28^null^ CD4^+^ T cells in extra-articular lesions in rheumatoid arthritis (182). Inflammatory CD56^+^ T cells have been reported in coronary artery disease, asthma, ulcerative colitis, and chronic hepatitis C disease (183–186). NKG2D^+^ CD28^null^ T cells have some tumor-promoting activity in experimental settings (187, 188) and as inflammatory mediators in Wegener’s granulomatosis, rheumatoid arthritis, juvenile-onset systemic lupus erythematosus, and celiac disease (189–192).

Many of these diseases have characteristic systemic upregulation of TNFα (193). We have shown that TNFα can directly block the *CD28* transcriptional initiator (65, 194). In a TNFα-rich environment, such as in the case of rheumatoid arthritis, we found that anti-TNF therapy prevents the TNFα-induced loss of CD28 on the residual CD28^+^ CD8^+^ and CD4^+^ T cells, but the numbers of CD28^null^ T cells remain the same (194). Whether or not TNFα induces the gain NK-related receptors has not yet been examined.

Interestingly, CD56^+^/NKG2D^+^ T cells also have beneficial effects in disease settings. Regulatory CD56^+^ CD28^null^ CD8^+^ T cells and NKG2D^+^ T cells have been reported in rheumatoid arthritis and in juvenile-onset systemic lupus erythematosus, respectively (195, 196). Similar NK-like T cell subsets appear to be normal components of regional host defense in the gut. They may have auxiliary antitumor effect and have been associated with antiviral immunity in the setting of allergies and chronic hepatitis B disease (197–200).

Such age-disproportionate emergence of senescent CD28^null^ NK-like T cells supports the provocative idea that premature senescence of T cells is a critical factor in the pathogenesis and clinical prognosis of chronic diseases of the young (201). These apparent beneficial and detrimental effects of certain NK-like T cell subsets among young patients, and the beneficial effects of similar cells during aging as described above, are consistent with the evolutionary concept of antagonistic pleiotropy (202). This concept posits that genes and biological pathways that are beneficial in the young may be detrimental in the old, and vice-versa. Therefore, a scientific challenge is to determine conditions in disease states of the young where CD28^null^ NK-like T cells might exert a pathogenic effect. It will be of similar interest to determine what drives the accumulation of beneficial senescent CD28^null^ NK-like T cells during the aging process.

## Conclusion: The Challenge of Harnessing Benefits of CD28^null^ NK-Like T Cells

The expression of NK-related receptors along clonal lineages of CD28^null^ T cells with aging clearly represents a reshaping or remodeling of the immune repertoire. T cell signaling through these receptors independent of the TCR also illustrates the emerging theme that cell senescence may not necessarily be synonymous with dysfunction. One scientific challenge is to determine what drives the induction of diversity of expression of NK-related receptors on T cells with advancing age. Another is to determine whether the TCR-independent effector function of NK-like T cells translates into vigorous immune defense and/or immune surveillance in late life. A corollary interest is a possible dual functionality of these T cells, namely, their ability to trigger a classic TCR-driven response, while triggering a complementary innate TCR-independent response mediated through the particular NK-receptor(s) they express. Plausibility of this dual function has been shown experimentally for the interaction between tumor cells and particular NK-like CD8^+^ T cell lines *in vitro* (203). An equal challenge is to elucidate the paradoxical age-disproportionate accumulation of NK-like T cells in disease states. Whether they represent cells involved in tissue repair or if they are true pathogenic effectors will be instructive into harnessing or dampening their effector function in disease settings. During the aging process, the most significant challenge is to determine how and why particular subsets of NK-like CD28^null^ T cells are closely linked to physical performance and cognitive ability. Dissecting these mechanisms will depend on the analyses of defined populations of the elderly, rather than continuing with the usual young-versus-old comparisons.

## Author Contributions

JM, PG, and AV drafted and edited the manuscript. JM and PG generated the figures. AV secured funding.

## Conflict of Interest Statement

The authors declare that the research was conducted in the absence of any commercial or financial relationships that could be construed as a potential conflict of interest.

## Abbreviations

3MS, average of three tests of the modified minimental examination; ADL, activities of daily living; GMFI, geometric mean fluorescence intensity; IFN, interferon; IL, interleukin; TCR, T cell receptor; TNF, tumor necrosis factor.
